# Validation of the Sinhalese Version of Brief COPE Scale for patients with cancer in Sri Lanka

**DOI:** 10.1186/s40359-022-00863-z

**Published:** 2022-06-20

**Authors:** Eranthi Weeratunga, Chandanie Senadheera, Manjula Hettiarachchi, Bilesha Perera

**Affiliations:** 1 Department of Nursing, Faculty of Allied Health Sciences, University of Ruhuna, Galle, Sri Lanka; 2 Department of Psychiatry, Faculty of Medicine, University of Ruhuna, Galle, Sri Lanka; 3 Nuclear Medicine Unit, Faculty of Medicine, University of Ruhuna, Galle, Sri Lanka; 4 Department of Community Medicine, Faculty of Medicine, University of Ruhuna, Galle, Sri Lanka

**Keywords:** Brief COPE, Cancer Coping skills, Reliability, Validity, Sri Lanka

## Abstract

**Background:**

Coping strategies play a vital role in cancer management and has been an integral part in the recovery process of cancer patients worldwide. Coping refers to specific efforts; both behavioral and psychological, that diminishes stresses emerged in cancer patients. This study evaluated the psychometric properties of the Sinhalese version of the Coping Orientation to Problems Experienced Inventory (S-BC) which was developed based on the Brief COPE scale  for cancer patients in Sri Lanka.

**Methods:**

The original Brief COPE is a self-administered tool with 28 items designed to measure coping methods used by people in stressful life events. It consisted of statements related to adaptive and maladaptive coping strategies. Forty patients with cancer who were registered at the Oncology ward, Teaching Hospital, Galle, Sri Lanka were included in the study. A cross-cultural adaptation of the Brief COPE was done using WHO guidelines. Reliability of the S-BC was assessed using test–retest and internal consistency procedures. The construct validity of the tool was assessed by performing exploratory factor analysis (EFA) and confirmatory factor analysis (CFA). Convergent and discriminant validity of the S-BC was tested using World Health Organization-Quality of Life-Brief scale (WHOQOL-BREF) and Centre for Epidemiological Studies-Depression scale (CES-D).

**Results:**

The mean (± *SD*) age of the sample was 61(± *12*) years, and 52.5% (n = 21) of the participants were men. Eighty percent (n = 32) of the participants were more than one year of treatment from diagnosing as a cancer patient. The test–retest reliability of the S-BC was 0.66, and the internal consistency of the S-BC was good (Cronbach’s alpha - 0.819). Both EFA and CFA revealed a structure comprised of seven factors. Such factors were Avoidance/Behavioral disengagement, Religious faith/Acceptance, Seeking support, Planning, Substance use/Venting, Self-blame and Active/positive coping. The scores of the adaptive coping of the S-BC was negatively and the scores of the maladaptive coping of the S-BC was positively correlated with the CES-D score.   The scores of the adaptive coping of the S-BC was  positively correlated with the total score of the WHOQOL-BREF questionnaire indicating the S-BC’s convergent and discriminant validity properties.

**Conclusion:**

The Sinhalese version of the Brief COPE is found to be a valid and a reliable measure to assess coping strategies used by  patients with cancer in Sri Lanka.

**Supplementary Information:**

The online version contains supplementary material available at 10.1186/s40359-022-00863-z.

## Background

Cancer is one of the major causes of death in the world and similar mortality trends of cancer have also been reported in Sri Lanka [1, 2]. Individuals diagnosed with cancer face life-threatening stressors. Patients react differently based on their personal and environmental conditions to their cancer condition by eliciting various coping responses [3]. ‘Coping’ is considered as a process by which people manage stress or attempt to manage stressful demands [4, 5]. Distress, depression, anxiety, hopelessness, fear, and aggression are found to be the consequences of stressors experienced by cancer patients. Such adverse consequences would create great impact on treatment, management, and control of the pathologies experience by cancer patients [6, 7].

Little attention has been paid by cancer patients as well as their family members on psychological and social aspects of the illness in the recovery process, and it was found that more personal and social support are needed for psychological and physical adjustment of cancer patients [6, 8]. Correct identification of coping strategies used by cancer patients is imperative to develop effective treatment and rehabilitation methods for cancer patients. Several self-report tools have been used to assess coping strategies of cancer patients worldwide [9, 10]. However, validated tool to measure coping strategies of cancer patients is not available in Sri Lanka; Way of Coping Checklist (WCCL) and the Brief COPE scale have been used to measure coping methods of undergraduates and parents of children with a chronic illness and developmental disability [11–16].

Carver et al. [8] developed the Coping Orientation to Problems Experienced (COPE) inventory and the Brief-COPE (B-COPE) based on theoretical foundations of coping methods. The full COPE comprises of 60 items, which has 15 conceptually distinct scales with four-items per scale [5]. To reduce the time required to complete the full scale, a Brief COPE (BC) scale (Additional file 1: Brief COPE scale, subscales and scoring methods) was introduced [8]. Carver’s scale has been validated in different countries and widely used to identify coping strategies among different groups of people including cancer patients; adolescents [17], offenders [18], textile workers [19], female breast cancer patients/survivors [20, 21], heart patients [22], and lung/gastrointestinal cancer patients [23].

A sample of breast cancer patients in Malaysia was used  to  examine the psychometric properties of the English version of the BC and it was found that the scale was valid and reliable for that population [21]. The Spanish version of the original BC was assessed using a sample of undergraduates in the America demonstrated reliability of the tool by  indicating  a high Cronbach's alpha for its internal consistency [23]. Previous EFA and CFA done on the BC scale had revealed structures with different number of factors including four [24], five [25], six [26], seven [3], and eight [27]. Such factor structures may be more sensitive to socio-cultural contexts in those populations. For example, a French version of BC was checked for the psychometric properties in a sample of French-Canadian breast cancer patients. Eight-factors were indicated by  factor analysis including factors  active coping, humor, religion, self-distraction, substance use, using emotional support, instrumental support and  disengagement [28]. Another study revealed a four-factor structure; social support, positive thinking, avoidance, and problem-solving for a French version of the BC scale [24]. A Spanish version of the BC scale had revealed a 12-factor structure which is similar to  the original BC [23]. Also like the original BC, 28-item/14 subscale was extracted in a Chinese study [29] with a Chronbach’s alpha value of 0.66 for the overall scale. In these 14 subscales Cronbach’s alpha varied between 0.70 and 0.80. Brasiliro et al. [30] had found a  three-factor structure and had termed the factors; religion, positive reframing, and distraction and external support.

It is very important and clinically meaningful to understand the coping strategies of  the patients with cancer for its management because of the  seriousness of this life-threatening illness, socio-economic burden of the illness and its severe side effects. Coping strategies may vary with the type of cancer and the stage of cancer. Assessing coping strategies in patients with cancer facilitates healthcare providers to get an insight of  the impact of coping on patients with cancer in different clinical and community settings which in turn facilitate them to provide effective treatments and needed welfare and social facilities [3, 20–22].

A culturally adapted version of Brief COPE scale would be more practical and useful to assess culture sensitive coping mechanisms of cancer patients in Sri Lanka. Such information is essential to improve the quality of cancer care services. The purpose of this study was to examine the psychometric properties of a culturally adapted Brief COPE scale for patients with cancer in Sri Lanka.

## Method

### Study setting and participants

A total of forty patients with cancer at the Radiotherapy Unit, Oncology Ward, Teaching Hospital, in Southern Sri Lanka were enrolled for the study [31–33]. Cancer patients were selected using the appointment register. Inclusion criteria included: having confirmed primary diagnoses as any type of cancer; planning for radiotherapy; able to understand local language Sinhalese and being able to provide informed consent with sufficient physical and mental stability. Subjects with any surgical problems other than cancer-related and in a critical state/end-stage of medical condition were excluded.

Medical officers usually plan radiotherapy for the patients after 14 days of their first visit. The purpose of the study was explained to the selected participants who were on  radiotherapy and then written informed consent was obtained. The participation in the study was completely on a voluntary basis.

### Measurements

Baseline characteristics of the cancer patients were obtained using an interviewer-administered questionnaire and the diagnosis cards of the patients. The information gathered  were age, gender, marital status, educational and employment status, type of cancer, and time since diagnosis (Additional file 2: Questionnaire). The  Centre for Epidemiological Studies-Depression scale (20 item-CES-D) [34] and the World Health Organization-Quality of Life-BREF scale (26 item-WHOQOL-BREF) [35] were administered along with the newa S-BC scale among 40 patients with cancer by the principal investigator (PI). Participants were informed to complete the S-BCCES-D, and WHOQOL-BREF which were previously validated in the Sri Lanka. Data were entered into a excel file (Additional file 3: Excel data sheet) without any identification information.

Sinhalese version of the Brief COPE scale (S-BC)—The original BC scale has 28 items and contains 14 subscales with two items on each scale, graded by a four-point Likert scale. The responses to the items were 1 = ‘I have not been doing this at all’, 2 = ‘I have been doing this a little bit, 3 = ‘I have been doing this a medium amount’ and 4 = ‘I have been doing this a lot. A higher score on the subscale items indicates a higher likelihood of using those strategies  by the respondents.

As previously mentioned, the BC has two subscale; adaptive coping and maladaptive coping and are shown  in the Additional file 1. The first eight subscales (16 items) were adaptive coping strategies, and the other six subscales (12 items) were maladaptive coping strategies [8]. A higher score indicates higher adaptive (score ranges from 16–64) or higher  maladaptive coping (score ranges from 12 to 48) practices. Cronbach’s alpha of the original BC was higher than 0.6 for 11 of its  14 subscales. In terms of usage, the BCscale has been widely used in different countries and has been shown good psychometric properties [18–20]. In this study a S-BC scale was used to collect data (Additional file 4: English version of S-Brief COPE scale).

The Centre for Epidemiological Studies-Depression scale (CES-D)—The CES-D is a 20-item, short and self-report scale, which was originally developed to assess depressive symptomatology in the  general population [34]. Each question has 04 responses from 0 (rarely or none of the time) to 3 (most or all of the time). The total score of the CES-D scale ranges from 0 (no depressive symptoms) to 60 (high level of depressive symptoms). The standard cut-off point that was used to identify those with elevated depressive symptoms was 16 or above. This scale has been validated in Sri Lanka [36].

The World Health Organization-Quality of Life-BREF scale (WHOQOL-BREF)—The WHOQOL-BREF is a 26-item quality of life (QoL) measurement scale and has four domains: physical, psychological, social, and environmental [35]. Higher values of the total scores indicate a higher level of QoL. The scale has been validated in Sri Lanka [37].

### Procedures

The BC was used for this study after  obtaining permission from the  original authors. Cross-cultural adaptation guidelines of Beaten et al. [38] and the World Health Organization (WHO) [39] were followed. These included  forward translation of the original version, expert panel discussion/synthesis, back-translation, cognitive interviewing, and pre-testing.

First, the forward translation of the original English version of the BC was done. by three independent experts who are fluent in both English and Sinhala language. The PI had independently recorded problems/issues during the forward translation. The multidisciplinary expert panel (including  an oncologists, clinical psychologist, clinician, a  behavioral scientist and a  senior lecture in nursing) discussion  was conducted to identify inappropriate statements and necessary changes were incorporated.

Again, three independent bilingual translators (different from the  the first group) back translated the Sinhalese version  into the English language. They were blind to the original tool. Validity checking was ensured by comparing the back-translated version and the original English version of BC. A preliminary version was developed after reviewing both forward and backward translations. A  cognitive interview was performed and focused on each statement to decide whether the wording is unclear, difficult to understand or answer, or to gain suggestions from participants to modify  the questions. Some wording modifications  were done  to make the tool  suitable for the Sri Lankan  culture. The new  scale was circulated among an expert committee to ensure clarity, ambiguity, and understandability of items in the questionnaire.

From the oncology wards, ten patients with cancer were enrolled for pre-testing (those who attended the pre-test were excluded from the main study). Patients were recruited according to the inclusion and exclusion criteria; participants were asked to state whether the items were readable and understandable. A modified version of the BC was found to be a practical tool that can be understood easily the patients with cancer. Finally, the panel of experts has ensured the content validity of the scale. They recommended that the S-BC should be used  as an interviewer-administered instrument due to many physical difficulties prevailing in  cancer patients (e.g., pain, discomforts) and variations in educational status.

The final Sinhalese version of Brief COPE (S-BC) scale was developed by incorporating face and consensual validity issues expressed by a group of health and research experts (relevance of each item of the scale in assessing coping according to patient’s life and adequacy of the scale to cover all relevant areas of coping were considered).

### Data quality

Several  steps were taken to increase the quality of the data gathered  because the target participants were cancer patients with various physical and psychological pathologies. Data collection was done by the PI personally. The purpose of the study and nature of the study was adequately explained to all patients and made them aware of the importance of giving true and valid responses in this survey.

### Data analysis

Data analysis was done using SPSS 25.0 (IBM statistics, Inc., Chicago). Descriptive statistics were used to illustrate baseline characteristics of cancer patients. The level of significance was taken as 0.05. The internal consistency of the S-BC was examined using Cronbach’s alpha coefficient for the  overall scale  of the S-BC and  for the subscales considering the accepted standard cut-off of 0.60 or above [26, 40].

The internal consistency of the S-BC was examined using Cronbach's alpha coefficient for an overall scale  of the S-BC and for subscales. The test–retest reliability was examined using intra-class correlation coefficient (ICC). (The PI administered the same questionnaire among the participants after two weeks of its first administration.). Pearson’s correlation coefficient was used to examine test–retest reliability [31].

Validity of the S-BC was checked using discriminant, , convergent , and construct validity. Pearson’s correlation coefficient was applied to check the discriminant and convergent validity of the S-BC scale [31]. Convergent validity was assessed by item-subscale correlation considering a higher correlation of each item with their respective subscale. Further, the  WHOQOL-BREF and CES-D scales were used to examine convergent and divergent validity. e.

Construct validity of the S-BC was assessed using EFA and CFA. The EFA was performed using Varimax rotation (Kaiser normalization/Kaiser–Meyer–Olkin (KMO)). Bartlett's Test of Sphericity should reach statistically significant result and commonalities coefficients should be high (> 0.6) to fulfil the conditions of this analysis [33]. The number of extracted components was determined by the Scree plot, percentage of variance explained by each component, number of Eigenvalues over one (Kaiser–Guttman rule), and by considering prior psychometric properties of Brief COPE. Items were considered representative of a component if their item loading was ≥ 0.40 and in the cross-loading items, the factor, which had a higher loading value, was taken as the respective factor [31].

CFA was evaluated using AMOS 23 software [41]. The root means square error of approximation (RMSEA) and comparative fit index (CFI) was examined. The cut-off values for acceptable model fit used for this study were: RMSEA ˃ 0.06 for a good fit; CFI ˃ 0.90 for an acceptable fit [42].

## Results

### Characteristics of the participants and descriptive statistics of coping strategies

A total of 40 patients with different cancer types participated in this study. Of these, 53.0% (n = 21) were males, 88.0% was married and 75.0% was employed at the time of the survey (Table 1). The mean (± SD) age of the sample was 61.03(± 11.70) years. The age of the participants ranged from 35 to 88 years. Response rate was 100%.

**Table 1:** Characteristics of the study participants (N = 40)

Characteristics/variables	Categories	N (%)
Age	< 60 Years	20 (50.0)
	˃ 60 Years	20 (50.0)
Gender	Male	21 (52.5)
	Female	19 (47.5)
Marital status	Married	35 (87.5)
	Unmarried/ single	5 (12.5)
Education	No schooling	5 (12.5)
	Primary education (grade 1–5)	9 (22.5)
	Secondary education (grade 6–12)	26 (65.0)
Employment	Employed	30 (75.0)
	Unemployed	10 (25.0)
Cancer types/locations	Head and neck cancer	2 (5.0)
	GI organs	12 (30.0)
	Lungs	1 (2.5)
	Bones	3 (7.5)
	Breast	3 (7.5)
	Prostate	1 (2.5)
	Lymph node	3 (7.5)
	Site unknown	15 (37.5)
Time since diagnosis	< 12 months	8 (20.0)
	˃ 12 months	32 (80.0)

N (%), frequency and percentage of patients

The mean (± SD) score of the adaptive coping was 37.50(± 8.14) and maladaptive coping was 17.10(± 2.44) (Table 2). The mean (± SD) S-BC scores of individual items are shown in Table 2). Of the Adaptive coping, the highest score was 6.87 ± 0.1.68 and it was for the item-1 ‘*Instrumental support’*; the lowest score was 2.00 ± 0.00 and it was for the item-8 ‘*Humor*’. In the maladaptive scale, the highest score was 5.08 ± 1.40 and it was for the item-1 ‘*Self-distraction’*; the lowest score was 2.05 ± 0.31 and it was for the items-5 and 6 ‘*Denial*’ and ‘*Behavioral disengagement’*.

**Table 2:** Cronbach’s alpha and mean values of S-BC scale/subscales

Subscales	Internal consistency (Cronbach’s alpha-α)		Mean ± SD
S-BC-adaptive coping		0.861	37.50 ± 8.14
	Instrumental support	0.85	6.87 ± .1.68
	Use of emotional support	0.70	6.68 ± 1.73
	Planning	0.48	5.30 ± 1.80
	Active coping	0.55	4.75 ± 1.69
	Religion	0.79	4.65 ± 2.00
	Acceptance	0.01	4.00 ± 1.03
	Positive reframing	0.07	3.25 ± 1.27
	Humor	–	2.00 ± 0.00
S-BC-maladaptive coping		0.396	17.10 ± 2.44
	Self-distraction	0.20	5.08 ± 1.40
	Venting	0.09	3.60 ± 1.21
	Substance use	0.52	2.20 ± 0.56
	Self-blame	0.06	2.12 ± 0.51
	Denial	–	2.05 ± 0.31
	Behavioral disengagement	1.00	2.05 ± 0.31

S-BC, Sinhalese Brief COPE scale; α, Cronbach’s alpha

### Cross-cultural adaptation of the S-BC

The S-BC  was found to be an easily comprehend, less time-consuming friendly tool and it  can be easily administered among cancer patients.. No significant difference between the original BC and and the S-BC was found. Participants could complete the tool within 15–20 minutes. Thus, the S-BC was found to be a culture sensitive and user-friendly measurement tool.

### Reliability of the S-BC

Due to the zero variance, three items were detached spontaneously from the reliability analysis (*item 18-I have been making jokes about it, item 28-I have been making fun of the situation, item 3-I have been saying to myself “this is not real”*). Then, retained 25-items were checked for reliability. Reliability of the scale (25 items) was found to be good (Cronbach’s alpha = 0.819); reliability of the adaptive scale (14 items) and maladaptive scale (11 items) were 0.861 and 0.396 respectively.

In test and retest scores, adaptive and maladaptive subscales of the S-BC had high Cronbach’s alpha; it was 0.793 and 0.788 respectively (Table 3). Test–retest reliability was higher for both adaptive and maladaptive scales (r ˃ 0.60, p < 0.01). Test–retest reliability measured with ICC between 1st and subsequent administration of adaptive and maladaptive scores reported higher ICC; adaptive: ICC = 0.65, 95% CI 0.43–0.80, p < 0.001, maladaptive: ICC = 0.65, 95% CI 0.42–0.79, p < 0.001. The results suggest that the S-BC scale has an acceptable reliability property.

**Table 3:** Reliability and test–retest reliability of S-Brief COPE scale—phase 1 and 2

Overall Cronbach’s alpha (25-items)	0.819	
	Adaptive	Maladaptive
	0.861	0.396

Time phase 1 and phase 2		
	1–2 Adaptive	1–2 Maladaptive
Cronbach’s alpha	0.793	0.788
Inter-Item correlation	0.659	0.651
Test re-test reliability	0.657^**^	0.651^**^

^**^p < 0.01

### Validity of S-BC

#### Criterion validity

Criterion validity of the scale was obtained (Table 4). Adaptive coping was inversely correlated with CES-D scores (discriminant validity) and maladaptive coping scores were positively correlated with CES-D scores (convergent validity). Adaptive coping, total QoL, and four domains of WHOQOL-BREF were positively correlated  with each other indicating convergent validity of the S-BC. Maladaptive coping was significantly and negatively correlated (discriminant validity) with total QoL, physical, and social QoL domains.

**Table 4:** Correlation between S-BC, CES-D, and WHOQOL-BREF

	S-BC subscales			
	Adaptive coping		Maladaptive coping	
	r	p	r	p
CES-D	− 0.362^*^	< *0.05*	0.349^*^	< *0.05*
Total QoL of *WHOQOL-BREF*	0.482^**^	< *0.01*	− 0.041	*0.80*
Physical domain of *WHOQOL-BREF*	0.506^**^	< *0.01*	− 0.117	*0.47*
Psychological domain of *WHOQOL-BREF*	0.338^*^	< *0.05*	0.040	*0.80*
Social domain of *WHOQOL-BREF*	0.328^*^	< *0.05*	− 0.030	*0.85*
Environmental domain of *WHOQOL-BREF*	0.415^**^	< *0.01*	0.029	*0.85*

r, correlation coefficient; CES-D, Centre for Epidemiological Studies-Depression scale; QoL, Quality of Life; WHOQOL-BREF, World Health Organization Quality of Life-Brief scale; p < 0.05* and p < 0.01**

#### Construct validity of S-BC

EFA and CFA were done. In EFA, some cross-loadings were reported (Table 5). The KMO measure of sampling adequacy suggests that data seems appropriate for factor analysis (KMO = 0.76). Bartlett’s Test of Sphericity reached statistical significance supporting that there is sufficient significant correlation in the data for confirmatory factor analysis (Chi square (190) = 408.27, p < 0.001).

**Table 5:** Factor loadings and cross-loadings emerging from EFA-S-BC

No	BC subscales	Items	Factor loadings						
			F1	F2	F3	F4	F5	F6	F7
*F1 Avoidance/Behavioral disengagement*									
6	I’ve been giving up trying to deal with it	**.994**							
8	I’ve been refusing to believe that it has happened	**.994**							
16	I’ve been giving up the attempt to cope	**.994**							
17	I’ve been looking for something good in what is happening	**.994**							
26	I’ve been blaming myself for things that happened	**.994**							
11	I’ve been using alcohol or other drugs to help me get through it	**.776**							
*F2 Religious faith/Acceptance*									
7	I’ve been taking action to try to make the situation better		**.549**			.438			
12	I’ve been trying to see it in a different light, to make it seem more positive		**.616**		.415				
14	I’ve been trying to come up with a strategy about what to do		**.591**		.418				
22	I’ve been trying to find comfort in my religion or spiritual beliefs		**.854**						
23	I’ve been trying to get advice or help from other people about what to do		**.627**						
24	I’ve been learning to live with it		**.802**						
25	I’ve been thinking hard about what steps to take		**.697**						
27	I’ve been praying or meditating		**.697**						
*F3 Seeking support*									
5	I’ve been getting emotional support from others			**.604**					
10	I’ve been getting help and advice from other people	.417	.427	**.550**					
19	I’ve been doing something watching TV, sleeping, or shopping			**.844**					
20	I’ve been accepting the reality of the fact that it has happened			− **.798**					
*F4 Planning*									
1	I’ve been turning to work or other activities to take my mind off things				**.846**				
2	I’ve been concentrating my efforts on doing something about the situation I’m in				**.857**				
*F5 Substance use/venting*									
21	I’ve been expressing my negative feelings					**.776**			
*F6 Self-blame*									
4	I’ve been using alcohol or other drugs to make myself feel better						− **.752**		
9	I’ve been saying things to let my unpleasant feelings escape				.465		**.591**		
*F7 Active/positive coping*									
13	I’ve been criticizing myself							**.768**	
15	I’ve been getting comfort and understanding from someone					.463		**.492**	
		Variance explained	24.97	42.83	52.13	61.37	67.37	73.35	78.68
		Eigen values	7.25	4.6	2.31	1.56	1.46	1.29	1.08
		Cronbach’s alpha	0.93	0.83	0.55	0.75	None	− 0.21	0.31

Extraction Method: Principal Component Analysis; Rotation Method: Varimax with Kaiser Normalization**;** Bold figures, items with mostly similar factor loadings

**Fig. 1:**
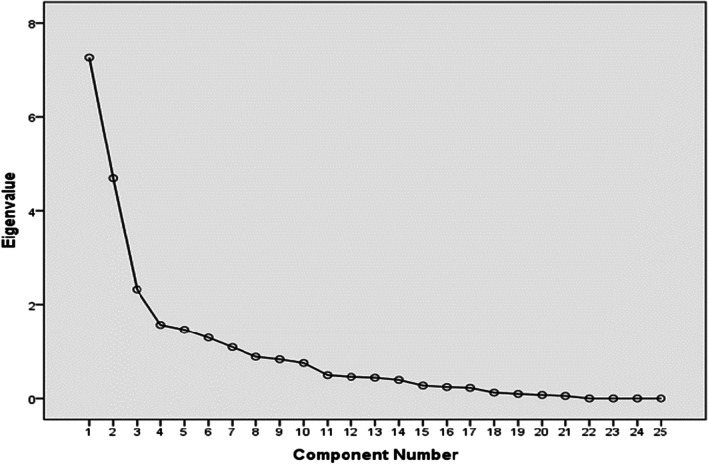
Scree plot of the S-BC

EFA extracted seven-factor model explaining cumulative variance of 78.68% (Table 5; Fig. 1. Scree plot). Three items in the original taxonomy of Carver et al. [8] were not included in the factor structure (items—3, 18, and 28) but the factors extracted represented entire subscales. The communalities of 25 items varied from 0.638 to 0.991 and factor loadings were varied from 0.415 to 0.994.

The 1^st^ extracted factor was loaded by six items of the original BC scale. Overall, this factor was termed as the “Avoidance/Behavioral disengagement”. The 2nd factor was loaded by eight items and was termed as “Religious faith/Acceptance”. The 3rd factor was loaded by four items and was termed as “Seeking support”. The 4th factor was loaded by two items and was termed as ‘Planning’. The 5th factor was loaded by one item and was termed as “Substance use/Venting”. The 6th factor was loaded by two items and was termed as “Self-Blame” and the last factor was loaded by two items and was termed as " Active/positive coping”.

**Fig. 2:**
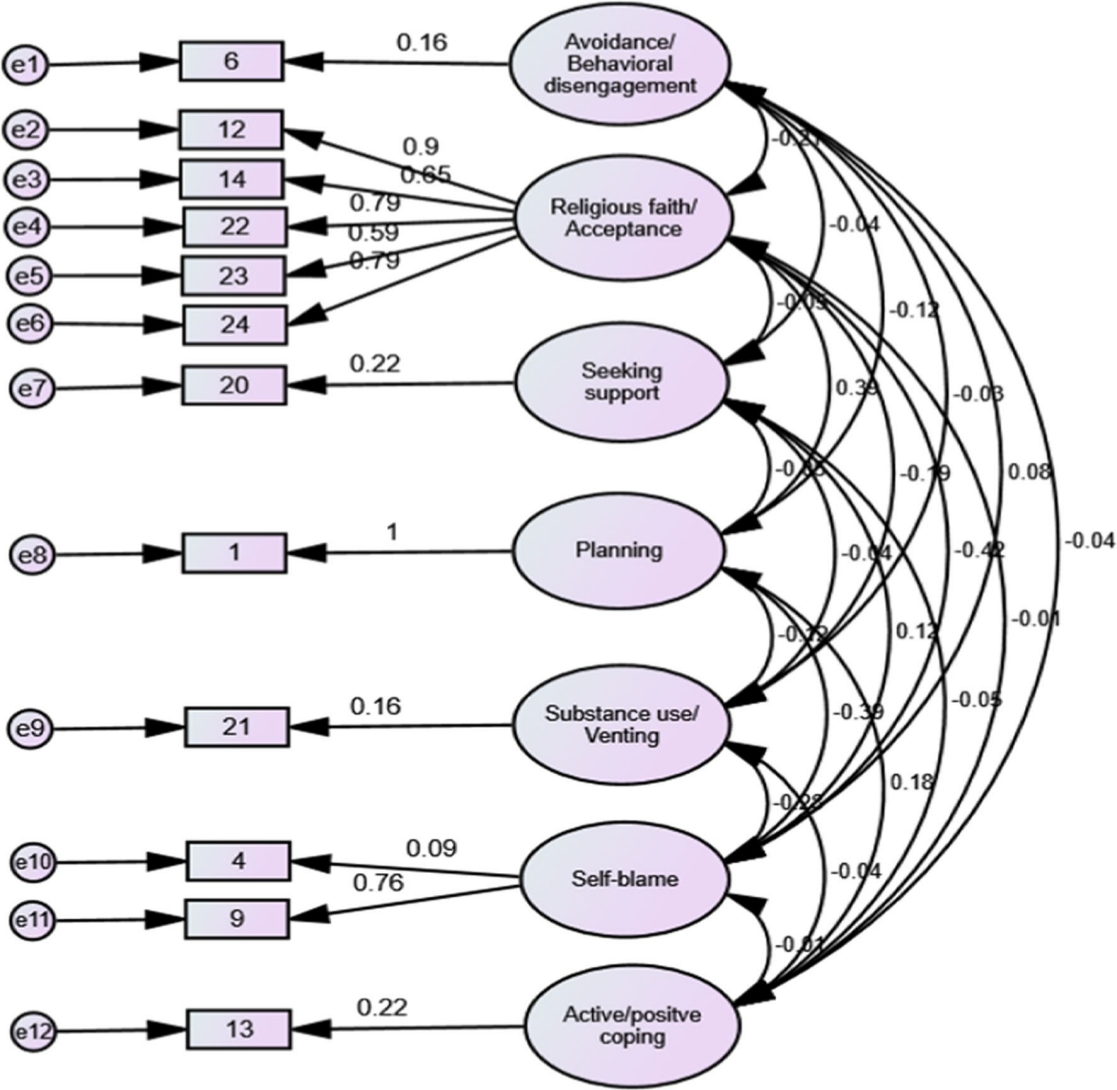
Path diagram of the model of the S-BC

CFA was performed (Fig. 2). Path diagram of the model of the S--BC was tested. The chi-square goodness-of fit p value was 0.428, and it indicates that there is no difference between the hypothesized model and the data. The CFI value was 0.985, greater than 0.90, indicating a good fit of the data. The RMSEA value was 0.025. The RMSEA value should be less than 0.08 to have a good fit. Therefore, the result indicate that this model has  a good fit for the data gathered.

## Discussion

This study examined the psychometric properties of a Sinhalese version of the Coping Orientation to Problems Experienced Inventory (S-BC). The S-BC is an adapted version of the original BC for cancer patients in Sri Lanka. It was found that the S-BC is a reliable and a valid tool to measure coping strategies used by cancer patients in Sri Lanka.

The S-BC was found to be a comprehensive, but a clear and a simple scale that can be used to measure coping strategies exhibit by cancer patients in the country. It has a good internal consistency and test–retest reliability. Cronbach's alpha of the S-BC was 0.819, a higher value compared to many other translated versions of the original BC [8, 27]. Adaptive coping items in the S-BC had a high Cronbach's alpha value (0.861) than that of maladaptive coping items (0.396). Three subscales in the adaptive coping (instrumental support, emotional support, and religion) and one subscale in the maladaptive coping strategies (behavioral disengagement) showed high Cronbach’s alpha values (> 0.7). Test–retest reliability of the S-BC was also high for both adaptive and maladaptive coping strategies. Therefore, the S-BC seems to be a reliable tool to measure coping strategies of cancer patients in Sri Lanka.

The S-BC showed a good criterion validity. As expected, the correlation between the adaptive coping scores of S-BC and the scores of CES-D was negative and the correlation between maladaptive coping scores of S-BC and the scores of CES-D was positive. The correlation between different subscales of the WHOQOL-BREF and adaptive coping scores of the S-BC was positive and no significant correlations were found between subscales of the WHOQOL-BREF and maladaptive coping scores of the S-BC.

In this study, seven-factors were extracted using the EFA. Those factors were termed as Avoidance/Behavioral disengagement, Religious faith/Acceptance, Seeking support, Planning, Substance use/Venting, Self-blame and Active/positive coping. A similar number of factors was obtained by Hagan et al. [3] and labeled as self-blame, acceptance, denial, emotional support, positive reframing, active, and behavioral disengagement. The French version of BC [28] was checked for its psychometric properties using a sample of French-Canadian breast cancer patients and eight factors were extracted: disengagement, self-distraction, active coping, using emotional support from husband/partner, using emotional support from friends, turning to religion, humor, and substance use. Another French study revealed a four-factor structure; social support, positive thinking, labeled avoidance, and problem-solving [24]. A Spanish version of the BC had revealed a 12-factor structure which is like the original BC structure [23]. In our study, it seems that several factors in the original scale tend to cluster together, and form border dimensions as observed by Miyazaki at el. [43].

Emotional and instrumental support items were included in a border factor termed seeking support in our study as seen in some other studies [26, 27]. CFA indicated good fit for the seven-factor model found in EFA. However, the weak and moderate correlations between the seven factors may indicate that cancer patients tend to employ only one or two coping strategies to cope with their distresses. Overall, the seven-factor model that this study observed seem to support the factor structure of the original BC.

This study has several limitations. The sample size was relatively small. It would be better if we could have had around 70 participants. Due to health conditions of this target population, we had to limit our sample to 40. Another limitation is that we have collected data from a single healthcare setting in Sri Lanka. Nevertheless, research efforts should be expanded on testing the S-BC in variety of cancer patients including possible other factors such as personality traits.

## Conclusion

The Sinhalese version of the BC scale was found to be a reliable and a valid tool to measure coping strategies among patients with cancer in the Sri Lanka. The S-BC can be used in clinical settings in Sri Lanka as a screening tool. At the international level, the study provides evidence of cross-cultural validation of the BC which is useful for comparative studies. Further research is needed to examine whether the factors identified in this research is related to demographic and other environmental factors such as gender, level of education and place of living. Most of the study participants had adaptive coping strategies and such information is useful for health authorities in the management of cancer patients.

## Supplementary Information

**Additional file 1**. Brief COPE scale, subscales and scoring methods.

**Additional file 2**. Questionnaire.

**Additional file 3**. Excel data sheet.

**Additional file 4**. English version of S-BC scale.

**Additional file 5**. ERC letter.

## Data Availability

The English version of BC scale, questionnaire, excel data sheet, English version of S-BC scale and ERC letter are included in this published article as Additional files 1, 2, 3, 4 and 5. The analyzed data during the current study are not openly available due to maintain the privacy of the participants’ identities but are available from the corresponding author on reasonable request.

## Abbreviations

Brief Coping/Brief COPE: Coping orientation to problems experienced inventory
SD: Standard deviation
CES-D: Centre for epidemiological studies–depression scale
WHOQOL-BREF: World health organization-quality of life-rief scale
BC: Brief cope
WHO: World health organization
S-BC: Sinhalese version of brief cope
ICC: Intra-class correlation coefficient
EFA: Exploratory factor analysis
KMO: Kaiser–meyer–olkin
PI: Principal investigator
THK: Teaching hospital karapitiya
QoL: Quality of life
SPSS: Statistical package for the social sciences
CI: Confidence interval
CFI: Comparative fit index
RMSEA: Root means square error of approximation
