# Supplementary material for: Validation of the Sinhalese Version of Brief COPE Scale for patients with cancer in Sri Lanka
Source: BMC Psychol. 2022 Jun 20;10:157. doi: 10.1186/s40359-022-00863-z (PMC9210691; doi:10.1186/s40359-022-00863-z)
Supplement: Supplementary file 1 — Additional file 1. Brief COPE scale, subscales and scoring methods. [file 40359_2022_863_MOESM1_ESM.docx]

**English version of 28-item Brief COPE scale**

1. I’ve been turning to work or other activities to take my mind off things.

2. I’ve been concentrating my efforts on doing something about the situation I’m in.

3. I’ve been saying to myself “this isn’t real.”

4. I’ve been using alcohol or other drugs to make myself feel better.

5. I’ve been getting emotional support from others.

6. I’ve been giving up trying to deal with it.

7. I’ve been taking action to try to make the situation better.

8. I’ve been refusing to believe that it has happened.

9. I’ve been saying things to let my unpleasant feelings escape.

10. I’ve been getting help and advice from other people.

11. I’ve been using alcohol or other drugs to help me get through it.

12. I’ve been trying to see it in a different light, to make it seem more positive.

13. I’ve been criticizing myself.

14. I’ve been trying to come up with a strategy about what to do.

15. I’ve been getting comfort and understanding from someone.

16. I’ve been giving up the attempt to cope.

17. I’ve been looking for something good in what is happening.

18. I’ve been making jokes about it.

19. I’ve been doing something to think about it less, such as going to movies, watching TV, daydreaming, sleeping, or shopping.

20. I’ve been accepting the reality of the fact that it has happened.

21. I’ve been expressing my negative feelings.

22. I’ve been trying to ﬁnd comfort in my religion or spiritual beliefs.

23. I’ve been trying to get advice or help from other people about what to do.

24. I’ve been learning to live with it.

25. I’ve been thinking hard about what steps to take.

26. I’ve been blaming myself for things that happened.

27. I’ve been praying or meditating.

28. I’ve been making fun of the situation.

**** Scoring system**- *Higher scores indicted higher adaptive and maladaptive coping.*

**Brief COPE scale with Adaptive, maladaptive subscales**

| Adaptive coping | Items (16) | Maladaptive coping | Items (12) |
| --- | --- | --- | --- |
| 1. Active coping | 2 and 7 | 9. Self-distraction | 1 and 19 |
| 2. Planning | 14 and 25 | 10. Denial | 3 and 8 |
| 3. Positive reframing | 12 and 17 | 11. Venting | 9 and 21 |
| 4. Acceptance | 20 and 24 | 12. Substance use | 4 and 11 |
| 5. Humor | 18 and 28 | 13. Behavioral disengagement | 6 and 16 |
| 6. Religion | 22 and 27 | 14. Self-blame | 13 and 26 |
| 7. Using Emotional support | 5 and 15 |  |  |
| 8.Using Instrumental support | 10 and 23 |  |  |
|  | |  | |
| Total score | 16-64 | Total score | 12-48 |
