# Supplementary material for: Validation of the Sinhalese Version of Brief COPE Scale for patients with cancer in Sri Lanka
Source: BMC Psychol. 2022 Jun 20;10:157. doi: 10.1186/s40359-022-00863-z (PMC9210691; doi:10.1186/s40359-022-00863-z)
Supplement: Supplementary file 2 — Additional file 2. Questionnaire. [file 40359_2022_863_MOESM2_ESM.docx]

**Questionnaire on psychosocial factors associated with quality-of-life patients with cancer**

Psychosocial factors associated with quality of life of patients with cancer are investigated.

This survey will take less than 10-15 minutes to fill. By filling the questionnaire your consent to participate in this research is given.

**Part 1: Give answers to the following questions**

| Q 1. | Age | ............................ |  |
| --- | --- | --- | --- |
| Q 2. | Gender | 1. Female ................ | 1. Male ........................ |
| Q 3. | Educational level | 1. No education | 1. 1-5 grade |
|  |  | 1. 6-10 grade | 1. Ordinary level |
|  |  | 1. Advanced level | 1. Diploma/Degree/Etc. |
| Q 4_1. | Occupation | 1. Professional | 1. Semi- professional |
|  |  | 1. Skilled worker | 1. Unskilled worker |
|  |  | 1. Laborer | 1. No occupation |
|  |  |  |  |
| Q 4_2 | Working status | 1. Doing currently. | 1. Not doing currently. |
|  |  | 1. Did not do any job. | 1. Retired/ pensionaries |
|  |  |  |  |
| Q 5. | Marital status | 1. Married. | 1. Unmarried. |
|  |  | 1. Separated. | 1. Divorced. |
|  |  | 1. Living along after death of spouse. | 1. Living together. |
|  |  |  |  |
| Q 6. | Time since diagnosis | 1. 6-12 months. | 1. 1-2 years. |
|  |  | 1. More than 2 years. |  |
|  |  |  |  |
| Q 7_1. | Removed surgically. | 1. Yes. | 1. No, |
|  |  |  |  |
| Q 7_2 | Type of cancer/ICD code | 5=C14Pharynx, 6=C15Oeso, 9=C20Rectum, 10=C21Anus, 12=C23Galle bladder,  18= C34Lung, | 20=C40Bones,  21=C50Breast, 26=C61Prostate,  33=C72Ear,  39=C97LN,  40=no/none) |
| Q 7_3 | Node | 1. Yes | 1. No |
| Q 7_4 | Metastasis | 1-C71=brain,  2-C2= tongue,  3-C15=esophagus,  4-C20= colon,  5-C22=liver,  6-C25=pancreases, | 7-C40=bones,  8-C70=spine,  9-C34=lungs,  10-C64=kidney,  11-C73=thyroid,  12-C50=breast,  13=none/no |

**Part II- Administration of scales- give answers to the following questions.**

**Brief COPE scale**

The Brief-COPE is a 28 item self-report questionnaire designed to measure adaptive and maladaptive ways to cope with a stressful life event; the scale can determine someone’s primary coping styles. Higher scores indicate higher adaptive and maladaptive coping.

*1= I haven’t been doing this at all, 2= A little bit, 3= A medium amount, 4= I’ve been doing this a lot*

| **No.** | **Coping strategies** | **1** | **2** | **3** | **4** |
| --- | --- | --- | --- | --- | --- |
| 8_1 | I’ve been turning to work or other activities to take my mind off things. |  |  |  |  |
| 8_2 | I’ve been concentrating my efforts on doing something about the situation I’m in. |  |  |  |  |
| 8_3 | I’ve been saying to myself “this isn’t real.” |  |  |  |  |
| 8_4 | I’ve been using alcohol or other drugs to make myself feel better. |  |  |  |  |
| 8_5 | I’ve been getting emotional support from others. |  |  |  |  |
| 8_6 | I’ve been giving up trying to deal with it. |  |  |  |  |
| 8_7 | I’ve been taking action to try to make the situation better. |  |  |  |  |
| 8_8 | I’ve been refusing to believe that it has happened. |  |  |  |  |
| 8_9 | I’ve been saying things to let my unpleasant feelings escape. |  |  |  |  |
| 8_10 | I’ve been getting help and advice from other people. |  |  |  |  |
| 8_11 | I’ve been using alcohol or other drugs to help me get through it. |  |  |  |  |
| 8_12 | I’ve been trying to see it in a different light, to make it seem more positive. |  |  |  |  |
| 8_13 | I’ve been criticizing myself. |  |  |  |  |
| 8_14 | I’ve been trying to come up with a strategy about what to do. |  |  |  |  |
| 8_15 | I’ve been getting comfort and understanding from someone. |  |  |  |  |
| 8_16 | I’ve been giving up the attempt to cope. |  |  |  |  |
| 8_17 | I’ve been looking for something good in what is happening. |  |  |  |  |
| 8_18 | I’ve been making jokes about it. |  |  |  |  |
| 8_19 | I’ve been doing something to think about it less, such as going to movies, watching TV, daydreaming, sleeping, or shopping. |  |  |  |  |
| 8_20 | I’ve been accepting the reality of the fact that it has happened. |  |  |  |  |
| 8_21 | I’ve been expressing my negative feelings. |  |  |  |  |
| 8_22 | I’ve been trying to ﬁnd comfort in my religion or spiritual beliefs. |  |  |  |  |
| 8_23 | I’ve been trying to get advice or help from other people about what to do. |  |  |  |  |
| 8_24 | I’ve been learning to live with it. |  |  |  |  |
| 8_25 | I’ve been thinking hard about what steps to take. |  |  |  |  |
| 8_26 | I’ve been blaming myself for things that happened. |  |  |  |  |
| 8_27 | I’ve been praying or meditating. |  |  |  |  |
| 8_28 | I’ve been making fun of the situation. |  |  |  |  |

- Phase 1 administration (P1)- P1Q8_1 to P1Q8_28
- Phase 2 administration (P2)- P2Q8_1 to P2Q8_28

**Center for Epidemiologic Studies Depression Scale (CES-D scale)**

Below is a list of the ways you might have felt or behaved. Please tell me how often you have felt this way during the past week.

0= Rarely or none of the time (less than1 day)

1= Some or a little of the time (1-2days)

2= Occasionally or a moderate amount of time (3-4 days)

3= Most or all of the time (5-7days)

| **No.** | **Statements** | **0** | **1** | **2** | **3** |
| --- | --- | --- | --- | --- | --- |
| 9_1 | I was bothered by things that usually don’t bother me. |  |  |  |  |
| 9_2 | I did not feel like eating; my appetite was poor. |  |  |  |  |
| 9_3 | I felt that I could not shake off the blues even with help from my family or friends. |  |  |  |  |
| 9_4 | I felt I was just as good as other people. |  |  |  |  |
| 9_5 | I had trouble keeping my mind on what I was doing. |  |  |  |  |
| 9_6 | I felt depressed. |  |  |  |  |
| 9_7 | I felt that everything I did was an effort. |  |  |  |  |
| 9_8 | I felt hopeful about the future. |  |  |  |  |
| 9_9 | I thought my life had been a failure. |  |  |  |  |
| 9_10 | I felt fearful. |  |  |  |  |
| 9_11 | My sleep was restless. |  |  |  |  |
| 9_12 | I was happy. |  |  |  |  |
| 9_13 | I talked less than usual. |  |  |  |  |
| 9_14 | I felt lonely. |  |  |  |  |
| 9_15 | People were unfriendly. |  |  |  |  |
| 9_16 | I enjoyed life. |  |  |  |  |
| 9_17 | I had crying spells. |  |  |  |  |
| 9_18 | I felt sad. |  |  |  |  |
| 9_19 | I felt that people dislike me. |  |  |  |  |
| 9_20 | I could not get “going.” |  |  |  |  |

The scoring of positive items is reversed. Possible range of scores is zero to 60, with the higher scores indicating the presence of more symptomatology.

- Phase 1 administration (P1)- P1Q9_1 to P1Q9_20
- Phase 2 administration (P1) - P2Q9_1 to P2Q9_20

**WHOQOL-BREF scale**

This assessment asks how you feel about your quality of life, health, or other areas of your life. Please answer all the questions. If you are unsure about which response to give to a question, please choose the one that appears most appropriate. This can often be your first response.

Please read each question, assess your feelings, and circle the number on the scale for each question

that gives the best answer for you.

| **No.** | **Statements** | **1** | **2** | **3** | **4** | **5** |
| --- | --- | --- | --- | --- | --- | --- |
| 10_1 | How would you rate your quality of life? | Very poor | Poor | Neither  poor nor  good | Good | Very good |
| 10_2 | How satisfied are you with your health? | Very  dissatisfied | Dissatisfied | Neither  satisfied nor  dissatisfied | Satisfied | Very  satisfied |
| 10_3 | To what extent do you feel that physical pain prevents you from doing what you need to do? | Not at all | A little | A moderate  amount | Very much | An extreme  amount |
| 10_4 | How much do you need any medical treatment to function in your daily life? | Not at all | A little | A moderate  amount | Very much | An extreme  amount |
| 10_5 | How much do you enjoy life? | Not at all | A little | A moderate  amount | Very much | An extreme  amount |
| 10_6 | To what extent do you feel your life to be meaningful? | Not at all | A little | A moderate  amount | Very much | An extreme  amount |
| 10_7 | How well are you able to concentrate? | Not at all | A little | A moderate  amount | Very much | Extremely |
| 10_8 | How safe do you feel in your daily life? | Not at all | A little | A moderate  amount | Very much | Extremely |
| 10_9 | How healthy is your physical environment? | Not at all | A little | A moderate  amount | Very much | Extremely |
| 10_10 | Do you have enough energy for everyday life? | Not at all | A little | Moderately | Mostly | Completely |
| 10_11 | Are you able to accept your bodily appearance? | Not at all | A little | Moderately | Mostly | Completely |
| 10_12 | Have you enough money to meet your needs? | Not at all | A little | Moderately | Mostly | Completely |
| 10_13 | How available to you is the information that you need in your day-to-day life? | Not at all | A little | Moderately | Mostly | Completely |
| 10_14 | To what extent do you have the opportunity for leisure activities? | Not at all | A little | Moderately | Mostly | Completely |
| 10_15 | How well are you able to get around? | Very poor | Poor | Neither poor nor good | Good | Very good |
| 10_16 | How satisfied are you with your sleep? | Very  dissatisfied | Dissatisfied | Neither  satisfied nor  dissatisfied | Satisfied | Very  satisfied |
| 10_17 | How satisfied are you with your ability to perform your daily living activities? | Very  dissatisfied | Dissatisfied | Neither  satisfied nor  dissatisfied | Satisfied | Very  satisfied |
| 10_18 | How satisfied are you with your capacity for work? | Very  dissatisfied | Dissatisfied | Neither  satisfied nor  dissatisfied | Satisfied | Very  satisfied |
| 10_19 | How satisfied are you with yourself? | Very  dissatisfied | Dissatisfied | Neither  satisfied nor  dissatisfied | Satisfied | Very  satisfied |
| 10_20 | How satisfied are you with your personal relationships? | Very  dissatisfied | Dissatisfied | Neither  satisfied nor  dissatisfied | Satisfied | Very  satisfied |
| 10_21 | How satisfied are you with your sex life? | Very  dissatisfied | Dissatisfied | Neither  satisfied nor  dissatisfied | Satisfied | Very  satisfied |
| 10_22 | How satisfied are you with the support you get from your friends? | Very  dissatisfied | Dissatisfied | Neither  satisfied nor  dissatisfied | Satisfied | Very  satisfied |
| 10_23 | How satisfied are you with the conditions of your living place? | Very  dissatisfied | Dissatisfied | Neither  satisfied nor  dissatisfied | Satisfied | Very  satisfied |
| 10_24 | How satisfied are you with your access to health services? | Very  dissatisfied | Dissatisfied | Neither  satisfied nor  dissatisfied | Satisfied | Very  satisfied |
| 10_25 | How satisfied are you with your transport? | Very  dissatisfied | Dissatisfied | Neither  satisfied nor  dissatisfied | Satisfied | Very  satisfied |
| 10_26 | How often do you have negative feelings such as blue mood, despair, anxiety, depression? | Never | Seldom | Quite often | Very often | Always |

- Phase 1 administration (P1) - P1Q10_1 to P1Q10_26
- Phase 2 administration (P1) - P2Q10_1 to P2Q10_26

***Thank you very much for your participation***

Please direct your inquires to

Ms. SMEB Weeratunga, Senior Lecturer, Department of Nursing, Faculty of Allied Health Sciences, University of Ruhuna (Tel- +94 77 22 58 519)
