# Supplementary material for: Validation of the Sinhalese Version of Brief COPE Scale for patients with cancer in Sri Lanka
Source: BMC Psychol. 2022 Jun 20;10:157. doi: 10.1186/s40359-022-00863-z (PMC9210691; doi:10.1186/s40359-022-00863-z)
Supplement: Supplementary file 4 — Additional file 4. English version of S-BC scale. [file 40359_2022_863_MOESM4_ESM.pdf]

Brief- COPE - Sinhalese version

Instructions:

The following questions ask how you have sought to cope with a hardship in your life. Read the statements and indicate how much you have been using each coping style.

1 = I haven't been doing this at all

2 = A little bit

3 = A medium amount

4 = I’ve been doing this a lot

	**Statements**	**1**	**2**	**3**	**4**
1	I’ve been turning to work or other activities to take my mind off things.				
2	I’ve been concentrating my efforts on doing something about the situation I’m in.				
3	I’ve been using alcohol or other drugs to make myself feel better				
4	I’ve been getting emotional support from others.				
5	I’ve been giving up trying to deal with it.				
6	I’ve been taking action to try to make the situation better.				
7	I’ve been refusing to believe that it has happened.				
8	I’ve been saying things to let my unpleasant feelings escape.				
9	I’ve been getting help and advice from other people.				
10	I’ve been using alcohol or other drugs to help me get through it.				
11	I’ve been trying to see it in a different light, to make it seem more positive.				
12	I’ve been criticizing myself.				

	Statements	1	2	3	4
13	I’ve been trying to come up with a strategy about what to do.				
14	I’ve been getting comfort and understanding from someone.				
15	I’ve been giving up the attempt to cope.				
16	I’ve been looking for something good in what is happening.				
17	I’ve been doing something to think about it less, such as going to movies, watching TV, daydreaming, sleeping, or shopping.				
18	I’ve been accepting the reality of the fact that it has happened.				
19	I’ve been expressing my negative feelings.				
20	I’ve been trying to find comfort in my religion or spiritual beliefs.				
21	I’ve been trying to get advice or help from other people about what to do.				
22	I’ve been learning to live with it.				
23	I’ve been thinking hard about what steps to take.				
24	I’ve been blaming myself for things that happened.				
25	I’ve been praying or meditating.				

THANK YOU !!!
