# Supplementary material for: Validation of the Sinhalese Version of Brief COPE Scale for patients with cancer in Sri Lanka
Source: BMC Psychol. 2022 Jun 20;10:157. doi: 10.1186/s40359-022-00863-z (PMC9210691; doi:10.1186/s40359-022-00863-z)
Supplement: Supplementary file 5 — Additional file 5. ERC letter. [file 40359_2022_863_MOESM5_ESM.pdf]

ETHICAL REVIEW COMMITTEE
FACULTY OF MEDICINE, UNIVERSITY OF RUHUNA
P.O. Box 70, Galle, Sri Lanka.

Chairman
Dr. G.H. Chandanie
MBBS, MD(Psych)
(+94) 0912246870,
0912234801 Ext.381

Convenor/Secretary
Dr. Ajith Nagahawatte
MBBS, Dip(Med.Micro), MD(Micro)
(+94) 0912246880,
0912234801 Ext.263

Fax: (+94) 0912222314

Members Ex officio
Dean
Dr. Sampath Gunawardena
MBBS, PhD

Director, Teaching Hospital, Karapitiya
Dr. Jayampathi Senanayaka

Members
Prof. Chithra Pathirana
B.Sc(Hon), PhD

Prof. Saman Wimalasundera
MBBS, DO, PhD

Dr. Bilesha Perera
B.Sc, M.Sc, PhD

Dr. Sathish Gunasinghe
MBBS, LLB, MS, FRCS

Dr. Isurani Ileyperuma
DVSc, PhD

Dr. Nayana Liyanaarachchi
MBBS, DCH, MD(Paed), MRCP

Dr. Menik Hettihewa
MBBS, PhD

Dr. K.A.C. Wickramaratne
MBBS, D.Path, MD(Haem)

Dr. P.V. de Silva
MBBS, MD(Comm.Med.)

Dr. A.J.P.M. Jayawardena
MBBS

Mrs. C.S. Samaranayake
Attorney-at-law, J.P.U.M.

04/12/2012

Ms. S M E B Weeratunga,
Allied Health Sciences Degree Program

Dear Ms Weeratunga,

Psychosocial factors associated with quality of life of cancer patients.
Ms. S M E B Weeratunga.

I am pleased to inform you that having reviewed the amendments submitted, ethical approval is given for the study pending ratification of the committee.

Thank you,

Yours sincerely,

Dr G H Chandanie

Copy : Convenor/ Secretary, Ethical Review Committee
